# Molecular markers of anti-malarial drug resistance in Lahj Governorate, Yemen: baseline data and implications

**DOI:** 10.1186/1475-2875-10-245

**Published:** 2011-08-21

**Authors:** Reem A Mubjer, Ahmed A Adeel, Michael L Chance, Amir A Hassan

**Affiliations:** 1 Genetics and Immunology, Department of Physiological Sciences, Faculty of Medicine and Health Sciences, Aden University, Yemen; 2 College of Medicine, King Saud University, Riyadh, Saudi Arabia; 3 Liverpool School of Tropical Medicine, Pembroke Place, Liverpool L3 5QA, UK

## Abstract

**Background:**

This is an investigation of anti-malarial molecular markers coupled with a therapeutic efficacy test of chloroquine (CQ) against falciparum malaria in an area of unstable malaria in Lahj Governorate, Yemen. The study was aimed at assessment of therapeutic response to CQ and elucidation of baseline information on molecular markers for *Plasmodium falciparum *resistance against CQ and sulphadoxine/pyrimethamine (SP).

**Methods:**

Between 2002 and 2003 the field test was conducted according to the standard WHO protocol to evaluate the therapeutic efficacy of CQ in 124 patients with falciparum malaria in an endemic area in Lahj Governorate in Yemen. Blood samples collected during this study were analysed for *P. falciparum *chloroquine resistance transporter gene (*pfcrt*)-76 polymorphisms, mutation *pfcrt-*S163R and the antifolate resistance-associated mutations dihydrofolate reductase (*dhfr*)-C59R and dihydropteroate synthase (*dhps*)-K540E. Direct DNA sequencing of the *pfcrt *gene from three representative field samples was carried out after DNA amplification of the 13 exons of the *pfcrt *gene.

**Results:**

Treatment failure was detected in 61% of the 122 cases that completed the 14-day follow-up. The prevalence of mutant *pfcrt *T76 was 98% in 112 amplified pre-treatment samples. The presence of *pfcrt *T76 was poorly predictive of *in vivo *CQ resistance (PPV = 61.8%, 95% CI = 52.7-70.9). The prevalence of *dhfr *Arg-59 mutation in 99 amplified samples was 5%, while the *dhps *Glu-540 was not detected in any of 119 amplified samples. Sequencing the *pfcrt *gene confirmed that Yemeni CQ resistant *P. falciparum *carry the old world (Asian and African) CQ resistant haplotype CVIETSESI at positions 72,73,74,75,76,220,271, 326 and 371.

**Conclusion:**

This is the first study to report baseline information on the characteristics and implications of anti-malarial drug resistance markers in Yemen. It is also the first report of the haplotype associated with CQR *P. falciparum *parasites from Yemen. Mutant *pfcrt*T76 is highly prevalent but it is a poor predictor of treatment failure in the study population. The prevalence of mutation *dhfr*Arg59 is suggestive of emerging resistance to SP, which is currently a component of the recommended combination treatment of falciparum malaria in Yemen. More studies on these markers are recommended for surveillance of resistance in the study area.

## Background

In Yemen the population at risk of malaria constitutes 81% of the total population, with an estimated one million cases in 2009 [1]. Most of the studies on anti-malarial drug efficacy that were carried out in the 1980s and the early 1990s were done in the southern parts of the country. These were mainly *in vivo *studies based on the standard WHO 7-day test to assess response of *Plasmodium falciparum *to chloroquine (CQ ) [2]. These studies reported no significant levels of CQ resistance during this period [3,4]. Starting from 2002, the revised WHO protocols were introduced for monitoring the therapeutic efficacy of anti-malarial drugs to *P. falciparum *in Yemen [5]. The present report gives an account of a therapeutic efficacy test on CQ conducted in 2002-2003 in Al-Musiemeer Hospital in Lahj Governorate and an investigation of molecular markers for CQ and sulphadoxine/pyrimethamine (SP) resistance in the same study samples. The findings establish baseline data on molecular markers of anti-malarial drug resistance, which could help in the surveillance of drug resistance in this area and in Yemen.

CQ resistance is associated with a point mutation at codon 76 of the *P. falciparum *chloroquine resistance transporter (*pfcrt) *gene, which is highly correlated with increased clinical CQ tolerance and treatment failure [6][7][8]. Other point mutations in *P. falciparum *multi-drug resistance gene 1 (*pfmdr1*) mainly N86Y, Y183F, S1034C, N1042D, and D1246Y, have also been shown to modulate CQ resistance [9]. In some areas, where CQ resistance was highly prevalent, studies have highlighted that withdrawal of CQ drug pressure may lead to a reversion to CQ-susceptible phenotypes as indicated by molecular marker studies [10]. Such a phenomenon might be missed if molecular prevalence surveys have not been conducted. Moreover, increasing evidence currently points to a possible role for both *pfcrt *and *pfmdr-1 *in resistance to other anti-malarial drugs [11,12]. A recent global meta-analysis concluded that both *pfcrt *and *pfmdr1 *polymorphisms are associated with chloroquine resistance, with the odds ratio (OR) of the *pfcrt *K76T mutation for therapeutic failure after chloroquine exceeding 7.0 at 28 days and 2.0 at day 14 [13].

Resistance of falciparum malaria parasites to antifolates is associated with mutation in dihydrofolate reductase (*dhfr*) and dihydropteroate synthase (*dhps*), which are enzymes involved in the parasite's folate synthesis [14-18]. Although (SP) alone is no longer recommended as a treatment regimen for falciparum malaria, it is still widely used in Yemen in combination with artesunate (AS) as first-line treatment of uncomplicated falciparum malaria. It remains essential that efficacy of the SP component should be closely monitored to ensure the effectiveness of the combination therapies that include it. Estimation of the prevalence of the molecular markers of SP and CQ resistance and validation of the association of mutations with resistance in different settings is needed for local policy guidance and for contributing to a global map for anti-malarial drug resistance [19].

## Methods

### Field therapeutic efficacy test

A therapeutic efficacy test was conducted according to the WHO protocol to evaluate CQ against falciparum malaria [5] between October 2002 and January 2003 in a rural hospital in Al-Musiemeer district, Lahj governorate in the south-east of Yemen. All febrile patients coming to the health centre during the study period were screened for parasitaemia and were subjected to a pre-treatment examination. Subjects were included if they were above six months of age, had a measured axillary temperature ≥ 37.5°C or a history of fever during the last 24 hours, had a positive *P. falciparum *mono infection and a parasite density of 1,000-200,000 parasites/μl of blood, if they were able to take oral medication with no history of intolerance to chloroquine, able to come for follow-up visits and have easy access to the health facility. An informed consent was obtained from all patients or guardians for children. Patients were excluded from the study if they had any sign of danger, severe malaria, severe malnutrition, concomitant other febrile illness that can interfere with the clear classification of the outcome, or if the patient was pregnant. A history of previous anti-malarial drug use was not an exclusion criterion; however, the information on previous use was carefully collected and recorded for each patient. Consecutive patients presenting to the health centre during the implementation of the study with symptoms suggestive of malaria and positive thick smear blood screening were enrolled in the study if they satisfied all the inclusion criteria. A hundred and twenty four cases were enrolled to the study. The sample size was calculated using Statcalc (*Epi info *Version 6). Since the treatment failure rate was unknown, and according the WHO guidelines, the *expected prevalence *of treatment failure (P) was assumed to be 50%. 10% *precision level *and 95% *confidence level *were used in calculation. The sample size required was 96 patients. An *expected follow-up loss *of 10% was added to minimize the possible bias due to it.

Data were entered in a special questionnaire that included all necessary study variables. To guide the treatment doses, patients were weighed on a reliably calibrated scale. Axillary temperature was recorded with a reliable, tested electronic thermometer. For microscopic blood examination, two blood slides were always taken for each patient, a thick film (for rapid staining and screening for parasitaemia while the patient is in attendance), and a thick and a thin film on the same slide for subsequent standard staining to calculate parasite density. Finger-prick blood was collected before treatment and during follow-up from each patient on FTA^® ^Classic Cards (Whatman^® ^BioSience) or ordinary Whatman No. 3 filter papers. The name of the patients, their numbers, day and date of collection were written on each filter paper. Each filter paper with blood was stored in individual plastic bag with silica gel and kept for subsequent molecular analysis. Slides of all patients were re-examined separately by two microscopists in the central laboratory of the Roll Back Malaria Programme.

WHO provided chloroquine (IDA-HOLLAND, Batch No.1636, expiry date October 2005), was administered in a total dose, 25 mg/kg body weight, under direct supervision, as a three-day course as follows: 10 mg/kg in Day 0, 10 mg/kg in Day 1 and 5 mg/kg in Day 2. During the three days, patients, especially children, were observed for one hour after administration of the drug. If they vomited the drug within 30 minutes of its administration, the drug was re-administered with the same dose, if vomited again the patient was excluded from the study. Patients were followed on an outpatient basis on Day 0, Day 1, Day 2, Day 3, Day 7, Day 14 and on any other day between the scheduled days in case of any health concern. The clinical condition, body temperature, parasitaemia were assessed at each visit. Blood samples were collected on filter papers on day 0, day 3, day 7, and on day 14 or the day of classification. The clinical and parasitological responses were classified according to the criteria of the standard WHO protocol into early treatment failure (ETF), late parasitological failure (LPF), late clinical failure (LCF) and adequate clinical and parasitological response (ACPR). Patients who were classified as a treatment failure were given the recommended dose of the second line treatment (SP). Two children developed signs of severe malaria (convulsions) during the follow-up period were given the first dose of parenteral quinine and taken urgently to the appropriate health facility.

### Molecular marker analysis

The molecular work started with testing three different methods for the extraction of DNA; namely the methanol fixation-heat extraction method, the FTA purification reagent and the QIAGEN kit extraction method from ordinary Whatman No. 3 filter papers and Whatman FTA^® ^Card. The three methods of extraction gave the same results when extracted DNA (from both ordinary Whatman No. 3 filter papers and FTA^® ^Cards) was used to amplify the *pfcrt *gene. It was decided to use the *Methanol-Fixation Heat Extraction *method because it was simple, effective, economic and feasible in poor resource countries like Yemen.

DNA was extracted from dried blood spots on filter paper using methanol-fixation/ heat extraction method as described by Plowe *et al *[17]. The PCR method developed by Djimde *et al *[20] was used to detect the *pfcrt*K76T mutation. It involves a nested PCR to amplify the region surrounding position 76 followed by restriction enzyme digestion to detect the presence of the mutant allele (Thr).

To detect the mutation S163R at *pfcrt *the protocol developed by Johnson [21], which is based on a nested PCR followed by restriction enzyme digestion was performed. DNA from the laboratory isolate K1AM that harbours the S163R mutation and DNA from 3D7 strain that lacks this mutation were used as controls, water was used as a negative control. To increase the sensitivity of the detection of the *dhfr*-59 polymorphism, two different PCR and restriction enzyme digestion protocols were used, one developed by Plowe *et al *[17] using *Bsr G*I enzyme for restriction digestion. The other method was that developed by Duraisingh *et al *[22] and uses *Xmn*I enzyme for restriction digestion.

A nested PCR reaction followed by restriction enzyme digestion was used to detect *dhps *polymorphism at codon 540. The method used was developed by Plowe *et al *[17]. The distinction between recrudescence and reinfections in treatment failure cases, and the genetic structure of the parasite population in the area were determined by nested family-specific PCR amplification of polymorphic regions of block 3 of *P*. *falciparum *antigen gene *msp-2 *as described by Snounou *et al *[23] and Magesa *et al *[24]. The primers *M2-OF *and *M2-OR*, were used in the first round amplification. Two separate second round reactions were performed, one reaction using a pair of FC27 family-specific primers (*M2-FCF *and *M2-FCR*) that detect the FC27-type variants, and the other reaction using a pair of 3D7/IC family-specific primers (*M2-ICF *and *M2-ICR*) that detect the 3D7/IC-type variants of MSP2.

Direct DNA sequencing of the *pfcrt *gene from three representative field samples was carried out by Lark Technologies. DNA was amplified using nested PCR using a number of primers that flank the 13 exons of the *pfcrt *gene (DJ Johnson personal communication).

### Ethical approval

This study was approved by the Ethical Committees of the Ministry of Public Health, the Faculty of Medicine & Health Sciences, Aden University in Yemen, and the Liverpool School of Tropical Medicine.

## Results

### In-vivo treatment outcomes

Of 644 febrile patients coming to the hospital, 225 (34.9%) were positive for *P. falciparum *mono infection, of which 124 (55.1%) were qualified for enrollment because they met all the inclusion criteria mentioned in the methods section. Patients of different age groups were enrolled (median 9.5, range 1-40). Of the122 cases that completed the follow-up, 48 (39.3%) had adequate clinical and parasitological response (ACPR), the remaining 74 (60.7%) were treatment failures. They were classified as Early Treatment Failures (ELF) 28 (23%), Late Clinical Failures (LCF) 16 (13.1%), and Late Parasitological Failures (LPF) 30 (24.6%).

### Predictors of chloroquine treatment failure

In this analysis, treatment outcomes were grouped into two groups; Adequate Clinical and Parasitological Response (ACPR) and Treatment Failure (TF). The TF group includes ETF+LCF+LPF. In univariate analysis, the following factors were found to be significantly associated with increased risk of CQ treatment failure: younger age (< 10 years), fever (≥ 37.5°C) and higher parasite density (≥ 25000 parasites/ul blood) at presentation (Table 1).

**Table 1:** Univariate analysis of some of the potential predictors of chloroquine treatment failure

Variable (no.)	Number of treatment failure	Odds ratio	95% CI	*p*-value
**Age (122)**				
**≥10 years (60)**	22	1.00	-	
**< 10 years (62)**	52	8.98	3.54-23.33	0.000

**Age (122)**				
**≥15 years (24)**	4	1.00	-	
**< 15 years (98)**	70	12.5	3.65-53.56	0.000

**Sex**		0.93	0.42-2.06	0.839

**Axillary temperature (122)**				
**< 37.5°C (79)**	42	1.00	-	
**≥37.5°C (43)**	32	2.56	1.06-6.30	0.022

**Parasite count (122)**				
**< 25000/ul blood (100)**	56	1.00	-	
**≥25000/ul blood (22)**	18	3.54	1.02-13.39	0.025

**Body weight (Kg)**		.891	.853-.931	0.000

Multivariate analysis using backward selection logistic regression confirmed that age less than 10 years is an independent predictor of CQ treatment failure (OR 8.7 95% CI 3.6-21.01), while the presence of fever and high parasite count lost their statistical significance. Body weight was dropped from multivariate analysis since it has a high correlation with age; Pearson correlation [25]*= 0.890, p < .001*. Among the treatment failure group the presence of fever (axillary temperature ≥ 37.5°C) at presentation, in addition to younger age (< five years) were found to be strong independent predictors of early treatment failure compared to late treatment failure (OR = 5.7 95% CI 1.96-16.58 and OR = 3.42 95% CI 1.04-11.21 for temperature and age respectively. Among late treatment failures, children less than 10 years of age were significantly at higher risk of being late clinical failures, compared to late parasitological failures, than older children and adults (*x^2 ^p value = .003*).

### Analysis of *pfcrt*76 point mutation

DNA was successfully amplified in all 112 pre-treatment samples. Of them, 109 (97.3%) carried the pure mutant T76 allele, 1 (0.9%) sample showed a mixed mutant and wild *pfcrt *genotypes, and 2 (1.8%) contained the pure wild K76 allele. In post-treatment samples DNA amplification was successful in 71 samples, 66 of which (93%) carried the pure mutant (T76) allele, 4 (5.6%) had mixed *pfcrt *genotype, and 1 (1.4%) carried the pure wild K76 allele. The *pfcrt *T76 was found in all post-treatment samples of patients who failed CQ treatment. Parasites carrying the wild K76 allele before treatment were not able to survive CQ treatment.

Analysis of *pfcrt*76 polymorphisms in the pre-treatment samples of 68 cases showed that 67 samples (98.5%) had the pure mutant T76 allele, one sample had mixed mutant and wild genotypes and none had the pure wild K76 allele. However, 42 of 44 (95.5%) of ACPR cases for whom DNA was available for analysis also appeared to have the pure mutant T76 allele in their pre-treatment samples. There was, therefore, no association between the presence of either the mutant T76 or the wild K76 allele and the treatment outcome; *Fisher exact p-value = 0.152 *(mixed genotype was added to the mutant). The prevalence of pre-treatment *pfcrt*76 polymorphisms in different categories of treatment outcome is shown in Table 2.

**Table 2:** Prevalence of *pfcrt*76 polymorphisms in pre-treatment samples in different categories of CQ treatment outcomes

	Treatment outcome				
**Type of pfcrt-76 polymorphism**	**ACPR (%)**	**ETF (%)**	**LCF (%)**	**LPF (%)**	**All (%)**

**Pfcrt-T76 mutant-type**	42 (95.5)	23 (100)	15 (100)	29 (96.7)	109 (97.3)

**Pfcrt-K76 wild-type**	2 (4.5)	0	0	0	2 (1.8)

**Pfcrt-T76/K76 mixed-type**	0	0	0	1 (3.3)	1 (0.9)

**Total (%)**	44 (100)	23 (100)	15 (100)	30 (100)	112 (100)

Sequencing the *pfcrt *gene of Yemeni parasites confirmed that Yemeni CQ resistant *P. falciparum *carry the old world (Asian and African) CQ resistant haplotype CVIETSESI at positions 72,73,74,75,76,220,271, 326 and 371.

### Effect of age on the association between *pfcrt*76 mutation and outcome

**Figure 1:**
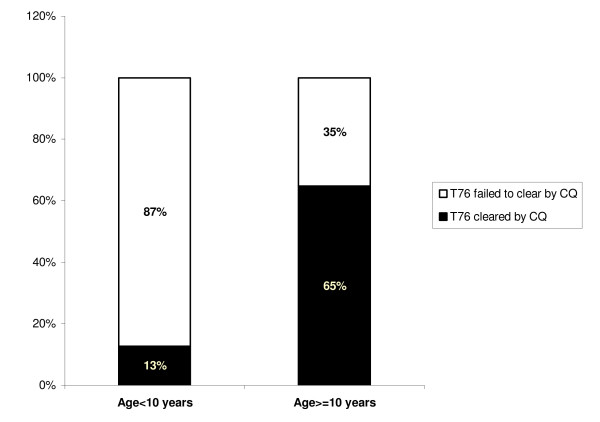
**Effect of age on the in-vivo clearance of parasites carrying pfcrt-76 mutation**.

The base-line prevalence of *pfcrt *T76 (and T76/K76) 110/112 (98.2%) was higher than that of clinical chloroquine resistance 68/112 (60.7%). To determine whether partial immunity developing with prolonged exposure to malaria contributed to the ability to clear infections caused by parasites carrying *pfcrt *T76, the proportion of infections by parasites carrying *pfcrt *T76 that cleared in children younger than 10 years of age were compared with the proportion of infections by parasites carrying *pfcrt *T76 that cleared in older children and adults. In the younger group, only 12.7 percent of 55 pre-treatment infections by parasites carrying the T76 mutation were successfully cleared by chloroquine, whereas in the older group 64.8 percent of 54 pre-treatment infections by parasites carrying T76 mutation were cleared by the drug, *X^2 ^p-value < 0.0001 *(Figure 1).

### Validation of the use of *pfcrt *-T76, as a molecular marker of *in vivo *chloroquine treatment failure

The sensitivity of the test, calculated by categorizing the treatment outcome into 2 categories; treatment failure (including ETF+LCF+LPF) and treatment success represented by ACPR, was found to be 100%, but the test was poorly specific (specificity = 4.5%, 95% CI = 0.0-10.7), The positive predictive value PPV was also low (PPV = 61.8%, 95% CI = 52.7-70.9). This result was expected due to the finding of high base-line prevalence of *pfcrt*T76 compared to clinical chloroquine treatment failure. Therefore, the genotype failure index GFI defined as the ratio of the prevalence of the resistant genotype (T76%) to the prevalence of chloroquine therapeutic failure both early and late (ETF%+LCF%+LPF%) was calculated and was found to be 1.6 (97.3/60.7) for all ages. Controlled for age, the GFI was found to be 1.2 (98.4/83.9) in children less than 10 years, whereas in older children and adults it increased to 2.8 (98.3/36.7).

### Screening for the presence of *Pfcrt*S163R

The presence of mutation *pfcrt*S163R was studied in 30 randomly selected pre-treatment samples of 42 samples of patients who, despite the presence of *pfcrt*K76T in their pre-treatment samples, did respond adequately to CQ (classified as ACPR with the in-vivo test). None of the 30 samples was found to carry the *pfcrt*S163R.

### Detection of *dhfr*-C59R and *dhps*-K540E

Using the method developed by Plowe *et al *[17] amplification of DNA to detect *dhfr*-C59R was performed in 119 pre-treatment samples. Amplification was successful in 80.7% (96/119) of the samples. Attempts to repeat and optimize the PCR in the 23 samples that failed amplification were not successful. In order to increase the sensitivity of the detection of *dhfr *polymorphism at codon 59, amplification was repeated in the failed 23 samples using an alternative PCR protocol developed by Duraisingh *et al *[22]. This resulted in successful amplification of only 13% (3/23) of the samples. Using the two PCR protocols for the detection of *dhfr*-C59R, amplification was successful in 83% (99/119) of the samples. Of the 99 successfully amplified samples 4 (4%) samples contained the *dhfr*-R59 mutant-type, one sample (1%) had the mixed-type C/R59 and 94 samples (95%) had the wild-type *dhfr*-C59. The mutant to wild genotype prevalence ratio (M/W) was calculated and found to be 0.05 (5/96). Amplification of DNA to detect the *dhps*-540 polymorphism was successful in all the 119 pre-treatment samples tested. All samples (100%) had the wild-type *dhps*-K540.

### Distinguishing recrudescence from reinfection using MSP 2

Twenty-four paired samples (pre-treatment and post-treatment) of patients who failed CQ treatment between day 7 and day 14 (LCF and LPF) were successfully amplified and thus included in this analysis. Seventy-one percent (17/24) of the samples had exactly the same alleles in both the primary sample and the recrudescent sample and they were classified as recrudescence. Twenty-one percent (5/24) of the samples had recrudescent plus new alleles in the recrudescent sample and were classified as indeterminate, and 8% (2/24) of the samples had only new alleles in the recrudescent sample and were classified as reinfection.

## Discussion

The present report gives the first account on anti-malarial molecular markers in Yemen, their frequency and their association with therapeutic response. Documentation of this baseline data is essential for surveillance of drug resistance and for global mapping of anti-malarial drug resistance. It is also the first report of the sequence of the *pfcrt *gene of Yemeni malaria parasites and the haplotype associated with CQR *P. falciparum *parasites from Yemen. Provision of such baseline information on the *pfcrt *gene from different geographical areas is essential for an understanding of the evolution of CQR in parasite populations [12].

The in vivo test described in this study indicates a high proportion of chloroquine treatment failure (61%). Subsequent therapeutic efficacy tests reported high levels of chloroquine resistance in other parts of Yemen and this lead to revision of the national malaria treatment policy, by switching the first-line treatment for uncomplicated falciparum malaria from CQ to the combination of SP+AS in 2005 (unpublished data, Dr H. Atta].

Multivariate analysis confirmed that young age is a strong, independent predictor of CQ treatment failure in this study. This could be explained by age being a surrogate of immunity in malaria endemic areas. The age association with treatment outcomes has been reported in areas of stable transmission e.g., Uganda [26], Ghana [27], as well as in areas of unstable transmission. In areas of unstable malaria in Sudan, Abdel-Hameed *et al *[28] reported that drug resistant cases of falciparum malaria were predominantly children, leading the authors to suggest the inclusion of children as a subgroup when testing efficacy in low transmission settings as they have a higher risk of therapeutic failure. Adam *et a*l [29] studied the factors that identify patients at risk of malaria treatment failure in an area of unstable malaria transmission in eastern Sudan. They analysed data from six clinical anti-malarial trials for uncomplicated falciparum malaria. They found that ability to clear CQ resistant parasites was significantly dependent on age, but not on the level of initial parasitaemia. Khalil *et al *[30] compared factors influencing parasite clearance after treatment in areas of different transmission intensity in Sudan and Tanzania. They found that parasite clearance was significantly associated with the initial level of parasitaemia (with P-values of 0.05 in Tanzania and 0.01 in Sudan) and with age (with P-values of 0.02 in Tanzania and 0.001 in Sudan). It seems that although previous observations indicated that the 76T and 86Y alleles play a role in the mechanism of CQ resistance, there is evidence that other factors, such as the level of parasitaemia when treated and age, are also important. The 76T and 86Y alleles could still be used as predictive markers for CQR, in non-immune individuals and low-transmission areas. The wild-type K76 was detected in only 1.8% of pre-treatment sample.

Talisuna *et al *[31] found that the prevalence of infections carrying the K76 wild genotype was more closely related to CQ resistance than that of the T76 mutated genotype. They suggested that the disappearance of infections with the wild genotype may be one of the last stages of the long process resulting in CQ resistance, and that drug pressure must be an important factor in this process. Drug pressure would probably select the T76 mutation and would consequently decrease the prevalence of the wild type (K76) [32]. The widespread use of CQ in this population might have highly selected for CQ resistant mutants in the parasite population. Bin Dajem and Al-Qahtani [33] have recently found mutant T76 in all 95 samples tested in an endemic area in southwest Saudi Arabia bordering Yemen.

In the present study, the prevalence of T76 (98%) was higher than the prevalence of *in-vivo *CQ treatment failure (61%). It was found that the T76 was present in all pre-treatment, as well as, post-treatment samples of patients who failed CQ treatment, and the only two pre-treatment samples that contained the wild K76 allele alone were belonging to patients who adequately responded to CQ, this indicates an absolute selection of the T76 mutation by the drug. The T76 genotype in pre treatment isolates was not predictive of in-vivo treatment failure. Similar finding of high prevalence of infection with theT76 genotype in pre-treatment isolates, which was not predictive of in-vivo failure level was observed in Sudan [34], Uganda [27], and in Loas [35]. These studies, like the present study, were conducted on patients seeking medical care, a selected group more likely to have taken anti-malarial drugs as compared to the general population [36,37]. Such results led to the conclusion that the presence of T76 may be necessary, but not sufficient, to predict *in vivo *treatment outcome in all patients. *In vivo *resistance may be influenced by a variety of factors, in addition to the K76T in *pfcrt*, including individual variations in drug absorption, pharmacokinetics, the underlying innate and acquired immune response, and the presence of additional mutations or compensatory changes in expression of other genes that may influence the level of resistance and ultimately the treatment outcome of patients infected with parasites that already display the K76T mutation. Giha *et al *[38] found evidence of clustering of treatment failure at level of individuals and households with differences in baseline immunity between the treatment failure prone individuals and treatment responders, suggesting an immune-mediated genetic susceptibility to treatment failure, as some of the tested polymorphisms showed trends but no significant association with treatment failures.

The presence of *Pfcrt*T76 in *P. falciparum *isolates in pre-treatment samples was poorly predictive of treatment outcome mainly because of the higher prevalence of the molecular marker for resistance, T76, than the in-vivo drug resistance due to the presence of T76 in pre-treatment samples of patients adequately responding to CQ. This finding has been noticed in nearly all studies [39]. This makes the application of this marker as a tool for surveillance challenging. Therefore, the ratios between the prevalence of the resistance genotype and the prevalence of the therapeutic failure (genotype-failure index GFI) were calculated [40]. The increase in the GFI with age in this study was consistent with Mali studies and reflects an acquired immunity and a higher proportion of older persons who cleared parasites with the CQ-resistant genotype when treated with CQ; it also reflects the intensity of transmission in the area. The role of immunity in clearance of resistant parasites was also observed in Burkina Faso, where children in ITC villages experienced an adequate clinical response more than children in non-ITC villages [41]. Induction of immunity through reduced exposure to malaria was explained by the frequent low density infection in children in ITC villages compared to frequent high density infections in children in non-ITC villages [41].

The finding of 5% of *dhfr-*R59 suggests that the prevalence of *dhfr *Asn-108 and *dhfr *Ile-51 mutations is higher than 5%. Previous studies showed that the prevalence of *dhfr- *C59 has been found to be lower than that of the other two *dhfr *mutations (*dhfr *Asn-108 and Ile-51) [42-44]. This might be explained by the idea of stepwise accumulation of *dhfr *mutations that follows the order Asn-108 → Ile-51 → Arg-59. The absence of *dhps*-E540 is similar to the finding of Khalil *et al *[45] in Khartoum in Sudan and similar to that of the Middle East were parasites are generally of wild-type *dhps *[46]. The stepwise process in selection of SP resistance might also explain the absence of *dhps*-E540 in the studied samples. According to different studies, it was found that, in areas of a high level of immunity, triple mutant *dhfr *with or without mutant *dhps *could be the main genetic determinant of SP treatment failure [43,47-49]. However, Alker *et al *[50] have found that *dhps-*437 and *dhps-*540 were strongly associated with SP treatment failure, while *dhfr-*59 was only weakly associated in eastern Democratic Republic of Congo.

Talisuna *et al *[51] suggested that *dhfr *codon 59 mutant to wild genotype ratio M/W is a simple and robust molecular marker that could be used for early detection of low SP treatment failure. In the present study, although not a population-based study, the M/W genotype ratio for the *dhfr *codon 59 was calculated to provide base-line information and help in monitoring the emerging SP resistance in Yemen. The ratio was found to be 0.05 (5/95) and according to Talisuna *et al *[51] this might predict < 10% SP treatment failure. A therapeutic efficacy trial of SP monotherapy was conducted in the study area in 2004 and found treatment failure of 5% [unpublished data, Dr. H. Atta]. The result of this study of the SP molecular markers indicated generally that the prevalence of the triple mutation indicated by *dhfr *Arg-59 was relatively low, 5%, whereas the *dhps *Glu-540 was very rare suggesting that the selection process had not reached *dhps*. However, eight years have passed since the study, and in this area where the people are aware that CQ is no longer effective in treating malaria and where SP is readily available even over the counter in pharmacies further selection of *dhfr *and *dhps *mutations might have rapidly taken place. Significant increase in the frequency of *dhfr-*59 and *dhps-*540 mutations was noticed in Sudan and Uganda over 3-4 year period [52,53]. This could compromise the efficacy of SP/AS combination, which is the current first-line treatment for falciparum malaria in Yemen. Preliminary results of therapeutic efficacy tests for the combination of SP+AS done in 2009-2010 indicate that it is still successful with the latest studies reporting 100% adequate clinical and parasitological response rates in two sites (Sharia-Almqarba in Odein), but showed a total failure rate of 2% in one site (Tor Bani Qais) [unpublished data, Dr H. Atta]. Close monitoring with molecular markers is needed to so that the identification of early markers of resistance will facilitate more widespread deployment of rational treatment policies that will retard the emergence of anti-malarial drug resistance.

## Competing interests

The authors declare that they have no competing interests.

## Authors' contributions

RAM: principal investigator, conducted the field work, molecular marker and sequencing tests, data analysis, wrote the manuscript. AAA: participated in establishment of the field work, data analysis, wrote the manuscript. MLC: Participated in establishment of the molecular and sequencing tests, data analysis, revised the manuscript. AAH: general supervision of the work, revising the manuscript. All authors read and approved the final manuscript.
